# Self-Similar Functional Circuit Models of Arteries and Deterministic Fractal Operators: Theoretical Revelation for Biomimetic Materials

**DOI:** 10.3390/ijms222312897

**Published:** 2021-11-29

**Authors:** Gang Peng, Jianqiao Guo, Yajun Yin

**Affiliations:** 1 Department of Engineering Mechanics, Tsinghua University, Beijing 100084, China; pg16@mails.tsinghua.edu.cn; 2 MOE Key Laboratory of Dynamics and Control of Flight Vehicle, School of Aerospace Engineering, Beijing Institute of Technology, Beijing 100081, China; guojianqiao@bit.edu.cn

**Keywords:** arteries, infinite-level structure, self-similar functional circuit, fractal admittance operators, biomimetic materials

## Abstract

In this paper, the self-similar functional circuit models of arteries are proposed for bioinspired hemodynamic materials design. Based on the mechanical-electrical analogous method, the circuit model can be utilized to mimic the blood flow of arteries. The theoretical mechanism to quantitatively simulate realistic blood flow is developed by establishing a fractal circuit network with an infinite number of electrical components. We have found that the fractal admittance operator obtained from the minimum repeating unit of the fractal circuit can simply and directly determine the blood-flow regulation mechanism. Furthermore, according to the operator algebra, the fractal admittance operator on the aorta can be represented by Gaussian-type convolution kernel function. Similarly, the arteriolar operator can be described by Bessel-type function. Moreover, by the self-similar assembly pattern of the proposed model, biomimetic materials which contain self-similar circuits can be designed to mimic physiological or pathological states of blood flow. Studies show that the self-similar functional circuit model can efficiently describe the blood flow and provide an available and convenient structural theoretical revelation for the preparation of in vitro hemodynamic bionic materials.

## 1. Introduction

Cardiovascular diseases have become one of the most dangerous factors for threatening human life [1,2,3]. According to investigation by the World Health Organization, about one-third of all deaths worldwide are caused by cardiovascular disease. Atherosclerosis is one of the most common cardiovascular diseases and has been extensively studied by epidemiologists [4,5]. High-risk vascular plaque (or vulnerable atheromatous plaque) is the focused object for medical detection and treatment among atherosclerotic diseases [6]. The dynamic reason for plaque formation is that long-term hypertension in the artery leads to decreased compliance of vascular fiber and eventual hardening. In order to explore the formation process of plaque and the blood flow obstruction caused by the plaque, appropriate arterial biomimetic materials are needed to be assembled for in vitro mimicry. The preparation of efficient biomimetic materials also depends on the structure model that accurately describes the arterial blood flow behavior.

Thanks to the advantages of systematic and straightforward simulation of arterial blood flow, the functional circuit model is an applicable hemodynamic structure model for the research of the causes and diagnosis of cardiovascular diseases, for example, that electrochemical impedance spectroscopy sensors based on the functional circuit model can be used for the 3-D mapping and detection of metabolically active atherosclerotic lesions [7]. Moreover, the model has been widely used to mimic the arterial circulation system’s diverse physiological and pathological hemodynamic behavior [8,9,10,11,12].

The functional circuit model is established based on the electrical-mechanical analogy method, where the factors that affect blood flow, such as arterial compliance, blood inertia, or viscosity, are analogized to electrical components. We can use the electrical response of the circuit network composed of these components to mimic the dynamic response of blood. Since the circuit has the function of simulating hemodynamics, we term the model as a functional circuit model (FCM).

**Figure 1:**
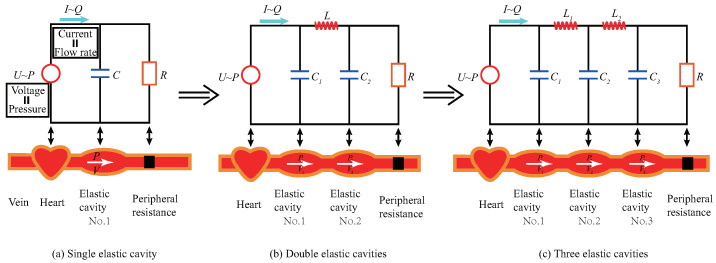
Classical finite-level elastic-cavity flow models of arteries and its analogous functional circuits: (**a**) single elastic cavity; (**b**) double elastic cavities; (**c**) three elastic cavities.

The FCM is also known as the elastic-cavity model, lumped parameter model, or Windkessel model. It is composed of standard electrical components such as capacitors, inductors, and resistors. Hales (1733) [13] first proposed and Frank (1899) [8] quantitatively described the simplest arterial systemic model. Frank [8] abstracts the arterial tube as an elastic cavity which is regarded as an electrical capacitive element. Here, the influence of arterioles and capillaries on blood flow is equivalent to a resistance element. With two electrical elements connected in parallel, a single elastic-cavity analogous circuit model is established (see Figure 1a). Inspired by that, subsequent researchers introduce more influencing factors and develop the multi-component model. For example, Burattini [9] and Stergiopulos et al. [10] notice blood inertia, which is analogized as inductance element, and establish a four-elements model. However, few types of factors on regulating blood flow can be explored.

Existing studies have shown that different structures, especially multilevel discrete patterns, can explain extraordinary phenomena in various disciplines. For example, fractional-order viscoelasticity response is induced by the hierarchical structures of ligaments and tendons fibers [14]. The spiking signal of neurons is related to the multilevel self-similar dendritic structures [15]. The vascular systems also have multilevel structures that gradually change from the aorta to capillaries, affecting the vascular physiological conditions [16,17,18]. We discover that the arterial vessels, like nerve fibers, also can be functionally abstracted into infinite-level structures. Retrospecting the traditional researches of the vascular elastic-cavity model, we can extract the idea that multilevel structures can control the behaviors of blood flow.

For instance, researchers have gradually explored numerically increased elastic-cavity models to accurately mimic the blood flow in the whole vessel system. Firstly, Frank’s classical single elastic cavity model is shown in Figure 1a, which includes only one elastic compartment representing the aorta [8,12]. Then, Goldwyn [19] and Baker et al. [20,21] analogized the aorta as the first elastic cavity and arteriole as the second one to establish a two-level four-element model, as shown in Figure 1b. Further, Abdolrazaghi et al. [11,12] separately considered the capillary arteries and established a three-level elastic-cavity model to research the blood flow of microcirculation, as shown in Figure 1c. Later, the elastic-cavity models with a finite number of components were developed to study the arterial behavior throughout the body. For example, Gul et al. [22] and Jager et al. [23] proposed finite elastic-cavity models to investigate the differences in blood flow due to the changes of arterial compliance, blood viscosity, and inertia at different segments. We have found that more and more levels of elastic cavities are introduced to simulate realistic blood flow. The multilevel functional structure pattern plays an important role in regulating blood flow. However, the traditional elastic-cavity models are all finite-level discrete structures, which cannot be theoretically equivalent to realistic blood flow.

As we know, blood vessels are continuous structures. Based on the Navier–Stokes equation, the classical continuum hemodynamic model is a solution for mimicking the blood flow in continuous vascular structures. Womersley [24,25], Morgan [26], Nichols [27], and Milnor et al. [28,29] have proposed that the blood flow in a consecutive arterial vessel can be regarded as the unsteady flow of the incompressible Newtonian fluid in a continuous elastic tube. However, the theoretical formulas of these continuum models are too tedious and require a mass of computational cost.

The selected elastic-cavity model with lumped parameters is the other appropriate and convenient method for simplifying the description of blood flow. According to the calculus approach [30], a continuous vascular structure can be treated as a combination of an infinite number of discrete elastic cavities with infinitesimal length. Therefore, the infinite-level elastic-cavity model can be developed to simply and equivalently mimic the blood flow within continuous vascular structures of arteries.

In physiology, arteries are divided into aorta and arteriole as the diameter of blood vessels decreases from large to small [31,32,33,34]. Based on the structural geometric size differences, Olufsen et al. [31,35,36] carried out a segmental structural tree model of arteries to reveal the differences in mechanical properties of different arterial parts. However, it is too complicated to describe the realistic continuous vascular system. Besides, blood vessels in different areas have different functions, and different influencing factors need to be considered. We mainly research two representative functional regions of the aorta and arteriole, respectively, and propose simple models with different electronic components to mimic the differences of aortic and arteriolar blood flow. The regulatory factors (vascular compliance, blood inertia, and blood viscosity) change with the blood flow from the proximal heart to the distal tissues. Apart from both being modulated by arterial wall compliance, blood flows in the aorta and arteriole are determined by blood inertia and viscosity, respectively. The reason is that the blood flow velocity is fast in the aorta, and the blood inertia is the main regulatory factor [9,37,38]. Relatively, arteriolar blood flow velocity slows down, and blood viscosity plays a major regulatory role [37,38].

Considering the influence of the structural pattern of the arterial elastic-cavity functional circuit on blood flow, this paper develops an infinite-level self-similar functional circuit model, which provides the structural theoretical revelation for the bioinspired design of in vitro hemodynamic mimic materials. The outline of this paper is as follows: In Section 2, the infinite-level self-similar functional circuit models of the aorta and arteriole are obtained from the abstracting infinite-level physical structures. The general expression of the fractal admittance operators which directly determine the response of blood flow is derived. In Section 3, two types of kernel functions in the convolution control equations of aortic and arteriolar blood flow are quantitatively described based on the fractal admittance operators. The differences and similarities of the two kernel functions themselves and the theoretical modulation mechanism of the harmonic blood-flow signal induced by them are analyzed. In Section 4, the advantages of the proposed ISFCM, the structural origins of the two functions, the theoretical revelation of the self-similar functional circuit model for biomimetic material design, the feasibility of shock disease study and selecting finite components retaining the self-similar structure form for the actual preparation of biomimetic hemodynamic materials, and the long-term goals are discussed. In Section 5, the conclusions of this study are summarized.

## 2. Models and Methods

### 2.1. Infinite-Level Physical Model and the Infinite-Level Self-Similar Functional Circuit Models of Arteries

There are three basic steps to building an infinite-level self-similar functional circuit model (ISFCM) for mimicking arterial blood flow.

**Figure 2:**
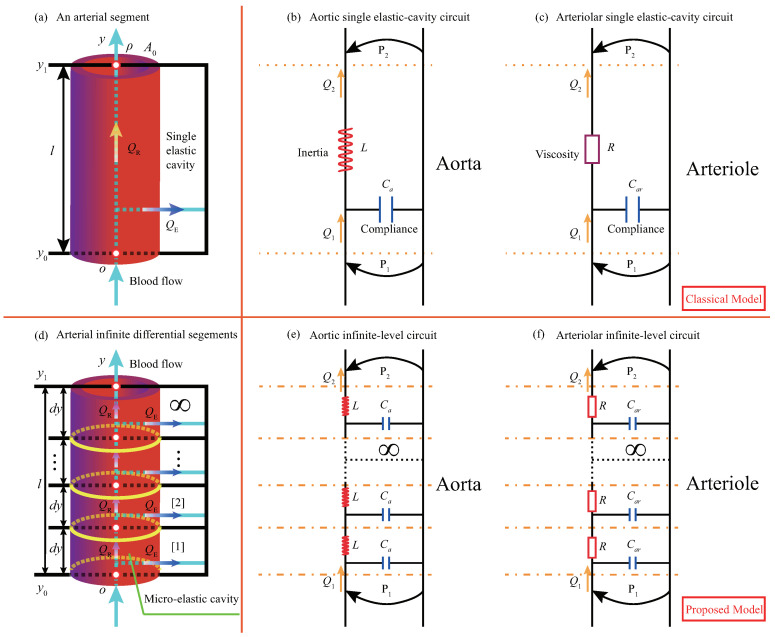
Aortic and arteriolar infinite-level elastic-cavity physic models expanded from the single one and their analogous circuits: (**a**) Physical structure of a single-level elastic cavity of an arterial vessel; (**b**,**c**) Analogous circuits of aortic and arteriolar single elastic cavity; (**d**) Physical structure of infinite micro-elastic cavities formed by micro-differentiation of a single elastic cavity; (**e**,**f**) Analogous circuits of infinite-level micro-elastic cavities of the aorta and arteriole.

**Figure 3:**
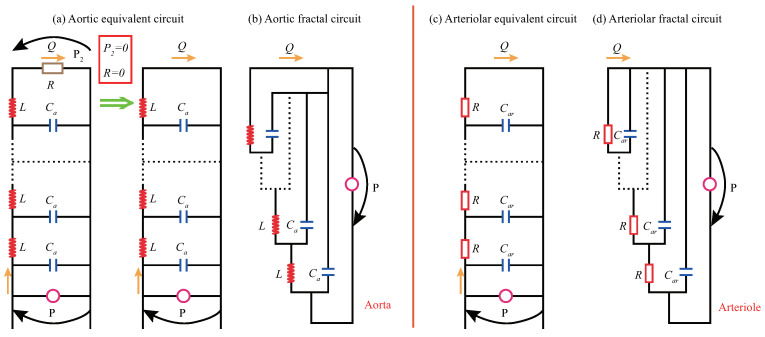
Infinite-level self-similar and fractal functional circuit, i.e., the infinite-level self-similar functional circuit model (ISFCM), of the aorta and arteriole, respectively: (**a**) Aortic equivalent circuit; (**b**) Aortic fractal functional circuit with infinite-level self-similarity; (**c**) Arteriolar equivalent circuit; (**d**) Arteriolar fractal functional circuit with infinite-level self-similarity.

- The first step is the mechanical-electrical analogy of the arteries. According to the classical elastic-cavity model, there are three main influencing factors of blood flow in the arteries. The factors can be analogized as three kinds of circuit elements:
  1. The flow induced by the longitudinal inertia of the blood flow is $Q_{I}$, which can be mimicked to the current on the inductance element *L*;
  2. The transverse flow caused by the compliance of vascular wall is $Q_{E}$, which can be equivalent to the current on the capacitor element *C*;
  3. The flow $Q_{R}$ caused by blood viscosity can be equivalent to the current on the resistance element *R*.
  The comparison relations of respective parameters are shown in Table 1.
  There are quantitative correlations in detail between the parameters of equivalent electrical elements and the physical parameters of arterial blood flow [38].
  1. The inductance *L* per unit length is proportional to the blood inertia (or density ρ) [11,38,39,40]:
    (1) $$L=\frac{c_{L}\rho }{\pi r^{2}};$$
  2. The capacitance *C* per unit length ($C_{a}$ and $C_{ar}$ for aorta and arteriole, respectively) is inversely proportional to the elastic modulus *E* of the tube wall [11,40,41] ($E_{a}$ and $E_{ar}$ for aorta and arteriole, respectively):
    (2) $$C=\frac{3\pi r^{2}}{2Eh};$$
  3. The resistance *R* per unit length is proportional to the blood viscosity μ [39,40]:
    (3) $$R=\frac{8c_{R}\mu }{\pi r^{4}},$$
  where, $c_{R}$ and $c_{L}$ are the constant coefficients, *r* is the radius of the vessel section, and *h* is the thickness of the vessel wall.
  Note that when we vary the electrical parameters, the corresponding blood flow parameters also change. Therefore, different physiological states of arterial vessels can be simulated by adjusting these electrical parameters. Primarily, atherosclerotic diseases are related to the parameter of vessel wall compliance. With different capacitors, arterial blood flow states under the different conditions of atherosclerosis can be mimicked.
- The second step is to abstract the physical model with an infinite number of micro-elastic cavities. Referring to the idea of mathematical localization analysis in calculus, the single-level elastic cavity can be micro-differentiated into a combination of infinite-level micro-elastic cavities (micro-segment dy). In this way, blood vessels can be regarded as a physical structure model with infinite-level micro-elastic cavities, as shown in Figure 2d.
  To simplify the problem, the arterial segments are treated as homogeneous. The elasticity of the tube wall, blood viscosity, and inertia are uniformly distributed along the longitudinal direction of the vessel. Thus, the physical parameters of wall compliance, blood viscosity, and inertia are constant in all micro-elastic cavities dy. Figure 2d is the physical model of blood flow in this paper.
- The third step is to abstract the self-similar analogous circuit, i.e., the ISFCM, from the physical model. According to the mechanical-electrical analogy, let dy→0, the infinite-level analogous circuits of the infinite-level micro-elastic cavities can be obtained, as shown in Figure 2e,f.
  In Figure 2, $P_{1}$ and $P_{2}$ are the input and output voltages or blood pressures, respectively. $Q_{1}$ and $Q_{2}$ are the input and output currents or blood flow rates, respectively. This paper mainly focuses on the input voltage $P_{1}$ and output current response $Q_{2}$ of the circuit under the regulation of respective functional components.
  In Figure 3, an input power supply voltage is introduced at the beginning of the equivalent arterial circuit. A resistor representing the peripheral resistance of blood vessels is added at the end to form a closed circuit. To analyze the regulation mechanism induced by the arterial basic analogous elements and combined structural patterns, we assumed that the electrical potential $P_{2}$ at the tail of the artery is zero, and the resistance *R* at the tail is ignored. Then, the equivalent circuit of the aorta is obtained, as shown in Figure 3a. Similarly, the arteriolar equivalent circuit can be derived, as shown in Figure 3c.
  Based on the equivalent transformation from Figure 3a,c into Figure 3b,d, it can be seen that the analogous circuit has self-similarity. Such infinite-level functional circuits are termed infinite-level self-similar functional circuits (ISFC), and for the reason that the circuits are utilized to mimic arterial blood flow behavior, we call it an infinite-level self-similar functional circuit model (ISFCM).
  On the other hand, the infinite-level self-similar functional circuit is also termed fractal functional circuit (FFC). The FFC in Figure 3 has infinite-level self-similar characteristics: for the aorta and the same with arteriole, each level of the circuit is composed of inductance and capacitance, and the next level infinitely repeats the previous level in a step shape. The FFCs of the arteriole and aorta have the same topological structures, but each level contains different components. Apart from capacitive elements, the aorta and arteriole contain inductance and resistance elements, respectively. Note that the FFC is a functional fractal with a specific physical analogous function and is different from general geometric fractal. The functional fractal is a kind of infinite-level self-similar functional structure without considering geometric scale invariance and fractal dimension.
  In the above analysis, the structure of finite-level models is stochastic. However, once the infinite-level circuit is abstracted, a new structural form can be derived, namely the infinite-level self-similar and functional fractal structural pattern shown in Figure 3. New concepts can be extracted with the infinite-level self-similar and fractal features, and new methods can be developed to describe the blood flow. Surprisingly, the research result can reach the unexpectedly exquisite and brief form.

### 2.2. Fractal Hypercell of the FFC in the ISFCM

**Figure 4:**
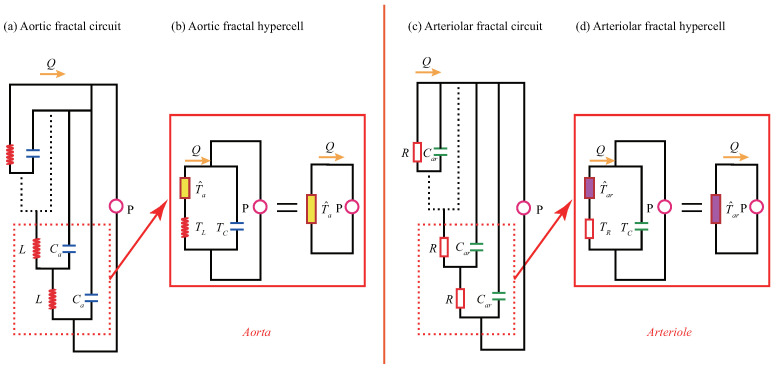
The fractal functional circuits (FFC) and fractal hypercells (FHC) of the aorta and arteriole: (**a**,**b**) the FFC and FHC of aorta, respectively; (**c**,**d**) the FFC and FHC of arteriole, respectively.

The minimum repeating unit in the FFC is termed fractal hypercell (FHC) [14], as shown in Figure 4. The earliest FHC concept was abstracted from the micro-nano structure of tendon fibers [14] and the analogous circuit of nerve fibers [15]. Now, the concept of FHC has been extended to the infinite-level self-similar functional circuit of the arterial blood flow.

Once the FHC is abstracted, we can simply study the relationship between the input and output signals of the analog circuit at the hypercell level and then clarify the dynamic responses of the ISFCM of the arteries.

### 2.3. Fractal Admittance Operators Describing the Time-Varying Blood-Flow Response of the Arterial ISFCM

We term the operator that determines the admittance characteristics of the FFC as the fractal admittance operator (FAO). Based on Heaviside’s operation of the calculus, the state differential equation of the circuit can be transformed into an algebraic equation of the operator, which means that the operator can be directly applied to describe the modulation mechanism of the whole circuit. Therefore, this paper studies the time-varying hemodynamic response of the arterial ISFCM by a simple but exquisite operator method.

The concept of FHC provides a carrier for introducing FAOs. The FAO on FHC is derived through the operator algebra method, which lays a foundation for the mathematical mode of the ISFCM.

The derivation of FAO should go through the following two steps.

**Figure 5:**
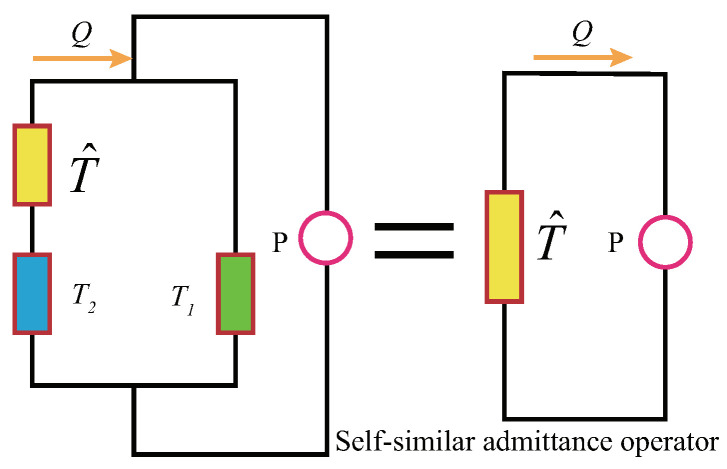
Unified fractal hypercell and fractal admittance operator of the aorta and arteriole.

- The first step is to express the admittance characteristics of the basic physical components included in the fractal circuit using an operator T(p):
  (4) i(t)=T(p)u(t),
  where i(t) and u(t) respectively represent the current and voltage in the time domain on the electrical element, *p* is the differential operator with respect to time, and T(p) is a function of the operator *p*. In operator algebra, differential operator *p* is defined as follows: If the function f(t) has continuous derivatives at t⩾0, then the differential operator acting on the function satisfies the relation [42]:
  (5) $$pf\left(t\right)=\frac{df(t)}{dt}.$$
  Corresponding to the differential operator, the integral operator *l* is defined as [42]:
  (6) $$l=\frac{1}{p}=p^{-1}.$$
  Thus, in the fractal circuits (see in Figure 3 and Figure 4), admittance operators of the three basic components can be explicitly expressed as follows.
  1. The admittance operator $T_{L}$ corresponding to the inductance element *L* is:
    (7) $$T_{L}=\frac{1}{Lp},$$
    which is a first-order integral operator.
  2. The admittance operator $T_{C}$ corresponding to the capacitance element *C* is:
    (8) $$T_{C}=Cp,$$
    which is a first-order differential operator. For aorta and arteriole, the capacitors are $C_{a}$ and $C_{ar}$, respectively.
  3. The admittance operator $T_{R}$ corresponding to the resistance element *R* is:
    (9) $$T_{R}=\frac{1}{R},$$
    which is a zero-order operator.
- The second step is to derive the admittance operator $\hat{T}$ of FHC, which directly determines the modulation mechanism of the ISFCM. Note that Figure 4b,d have the same topology and can therefore be represented uniformly as Figure 5.
  The meaning of operators $\hat{T}$, ${\hat{T}}_{1}$, and ${\hat{T}}_{2}$ is as follows. For the aorta,
  (10) $$\hat{T}={\hat{T}}_{a},T_{1}=T_{C},T_{2}=T_{L};$$
  and for the arteriole,
  (11) $$\hat{T}={\hat{T}}_{ar},T_{1}=T_{C},T_{2}=T_{R}.$$
  In Figure 5, on the whole structural pattern, the combination of the left fractal hypercell and the basic element at the same level is a fractal hypercell again at the next level, and their admittance operators are both equivalent to $\hat{T}$ (namely, fractal admittance operator), which is determined by the self-similar structure. The infinite-level self-similarity and equivalence in Figure 5 provide the following quadratic algebraic equation of the operator with one variable satisfied by the fractal admittance operator $\hat{T}$:
  (12) $${\hat{T}}^{2}-T_{1}\hat{T}-T_{1}T_{2}=0.$$
  Solving the equation, the general algebraic expression of the FAO can be written as:
  (13) $$\hat{T}=\frac{T_{1}\pm \sqrt{T_{1}^{2}+4T_{1}T_{2}}}{2}.$$
  The result is the theoretical basis for directly describing blood flow by ISFCM. The form is very similar to those of ligaments/tendons and nerve fibers [14,15].

## 3. Results

### 3.1. Hemodynamic Control Equations Characterized by FAOs in Aortic and Arteriolar ISFCM

In the aorta, the admittance operator of inductance element (Equation (7)) and capacitance element (Equation (8)) is substituted into the general expression of FAO (Equation (13)) and then the expression of aortic FAO can be obtained:

(14) $${\hat{T}}_{a}=\frac{C_{a}p\pm C_{a}\sqrt{p^{2}+4/\tau_{a}^{2}}}{2},$$

where $\tau_{a}=\sqrt{LC_{a}}$ represents the characteristic time of aortic blood flow determined by the wall compliance and blood inertia [43].

The total blood-flow response of the arterial FFC of the ISFCM depends on the FAO, and its governing equation can be written below:

(15) $$i\left(t\right)=\hat{T}\left(p\right)u\left(t\right).$$

Therefore, if the arterial input voltage signal u(t) is known, the output current signal i(t) can be easily obtained by modulation of the FAO $\hat{T}$.

Then, by substituting the algebraic expression Equation (14) of the aortic FAO into Equation (15) above, the aortic hemodynamic equation can be obtained:

(16) $$i_{a}\left(t\right)=\left(\frac{\pm C_{a}\sqrt{p^{2}+4/\tau_{a}^{2}}+C_{a}p}{2}\right)u_{a}\left(t\right).$$

In arteriole, similarly, the admittance operator of resistance element (Equation (9)) and capacitance element (Equation (8)) are substituted into the general expression (Equation (13)) and then the FAO expression of arteriole can be derived:

(17) $${\hat{T}}_{ar}=\frac{C_{ar}p\pm C_{ar}\sqrt{p^{2}+4p/\tau_{ar}}}{2},$$

where $\tau_{ar}=RC_{ar}$ represents the characteristic time of arteriolar blood flow determined by wall compliance and blood viscosity.

At the same time, the algebraic expression of the arteriolar FAO in Equation (17) was substituted into Equation (15) to obtain the arterial blood-flow dynamics equation:

(18) $$i_{ar}\left(t\right)=\left(\frac{\pm C_{ar}\sqrt{p^{2}+4p/\tau_{ar}}+C_{ar}p}{2}\right)u_{ar}\left(t\right).$$

### 3.2. Apparent Fractional-Order Differential Characteristics of Blood Flow Regulated by FAOs

The FAO $\hat{T}$ (Equation (13)) contains the quadratic radical $\sqrt{T_{1}^{2}+4T_{1}T_{2}}$. That seems to indicate that the FAO $\hat{T}$ has the fractional differential property of order 1/2 [14,15]. In other words, $\hat{T}$ should be a fractional operator of order 1/2. Yet the following analysis shows that this is only a representation.

The order analysis of the aortic FAO is as follows. According to fractional calculus, quadratic radical operator $\sqrt{p+A}$ has exponentially weighted modulated fractional differential form [15]. The fractional differential expression of Riemann–Liouville form is introduced, which can be written as:

(19) $$\sqrt{p+A}f\left(t\right)=\left\{e^{-At}p^{0.5}e^{At}\right\}f\left(t\right){=}_{0}^{RL}D_{t}^{0.5,A}f\left(t\right),$$

where *A* represents the numerical operators, which are operators satisfied by the definition of convolution operation [42]; ${}_{0}^{RL}D_{t}^{0.5,A}$ represents the 0.5-order exponential modulated Riemann–Liouville fractional differential. It can be seen that the quadratic radical operator $\sqrt{p+A}$ has the fractional differential property of order 0.5, which is the algebraic value.

However, in the algebraic expression of the aortic FAO, the radical contains the quadratic power of the differential operator $p^{2}$, which cancels the fractional order of 0.5. At this point, the blood-flow control Equation (16) of the aortic ISFCM can be rewritten into the Riemann–Liouville fractional differential definition form,

(20) $$i_{a}\left(t\right)={\hat{T}}_{a}u_{a}\left(t\right)=\frac{C_{a}}{2}\left[\pm_{0}^{RL}D_{t}^{1,4/\tau_{a}^{2}}+D_{t}^{1}\right]u_{a}\left(t\right).$$

It can be seen that ${\hat{T}}_{a}$ finally behaves as first-order integer exponential modulated differential property. We call this kind of FAO an “apparent fractional-order operator”, which appears to be of fractional order but has integer order in nature.

A generalized form of Equation (19) also has the fractional-order expression defined by Riemann–Liouville:

(21) $$\begin{array}{cc} \sqrt{p+A}\sqrt{p+B}f\left(t\right)\; & =\frac{e^{-At}}{\pi }\frac{d}{dt}\int_{0}^{t}\frac{e^{(A-B)\eta }}{{\left(t-\eta \right)}^{0.5}}\left[\frac{d}{d\eta }\int_{0}^{\eta }\frac{e^{B\xi }}{{\left(\eta -\xi \right)}^{0.5}}f\left(\xi \right)d\xi \right]d\eta \\ \; & {=}_{0}^{RL}D_{t}^{0.5,A}\left[{}_{0}^{RL}D_{t}^{0.5,B}\right]f\left(t\right). \end{array}$$

It has a double 0.5-order modulation-type fractional differential property. Similarly, it can also be regarded as a kind of “apparent fractional-order operator”.

For the aortic FAO, imaginary number decomposition also can be introduced:

(22) $$\frac{4}{\tau_{a}^{2}}=-\frac{2i}{\tau_{a}}\cdot \frac{2i}{\tau_{a}},$$

where *i* is the imaginary numerical operator.

Therefore, the aorta hemodynamic equation was rewritten in another dual 0.5-order modulated Riemann–Liouville apparent fractional-order differential form:

(23) $$i_{a}\left(t\right)={\hat{T}}_{a}u_{a}\left(t\right)=\frac{C_{a}}{2}\left[\pm_{0}^{RL}D_{t}^{0.5,2i/\tau_{a}}\left[{}_{0}^{RL}D_{t}^{0.5,-2i/\tau_{a}}\right]+D_{t}^{1}\right]u_{a}\left(t\right).$$

Similarly, the order of the arteriolar FAO ${\hat{T}}_{ar}$ also has the characteristics of apparent fractional order because that ${\hat{T}}_{ar}$ also contains the radical of the operator:

(24) $$\sqrt{C_{ar}^{2}p^{2}+4C_{ar}p/R}=C_{ar}\sqrt{p(p+4/\tau_{ar})}.$$

According to Equation (21), the blood flow control Equation (18) of the arteriolar ISFCM can also be rewritten in the form of dual 0.5-order modulated Riemann–Liouville apparent fractional-order differential expression:

(25) $$i_{ar}\left(t\right)={\hat{T}}_{ar}u_{ar}\left(t\right)=\frac{C_{ar}}{2}\left[\pm_{0}^{RL}D_{t}^{0.5}\left[{}_{0}^{RL}D_{t}^{0.5,{\bar{\tau }}_{ar}}\right]+D_{t}^{1}\right]u_{ar}\left(t\right),$$

where, ${\bar{\tau }}_{ar}=4/\tau_{ar}$.

In short, the FAOs of the aorta and arteriole have the characteristics of apparent fractional-order differential, which means that the blood-flow response of the ISFCM has the apparent fractional-order differential characteristic. Obviously, the differential order of the FAO depends not only on the fractional order of the radical in the algebraic domain, but also on the physical properties of the containing electrical elements or blood factors.

### 3.3. Gaussian-Type Convolution Modulation Kernel Function in Aortic FAO of ISFCM

The focus of Section 3.1 is to derive the algebraic expression of FAO rather than to provide a profound interpretation itself. We clarify that the FAO affecting the arterial blood flow has the form of convolution regulation in this section. It is found that the FAO contains the special function as the convolution kernel.

In operator algebra, operators also can be expressed as modulation kernel function form with convolution definition [42]. This section is devoted to exploring the kernel function of aortic FAO ${\hat{T}}_{a}$.

For the convenience of analysis, we decomposed the Equation (14) of the aortic FAO into the sum of the basic term and the modulation term:

(26) $${\hat{T}}_{a}=T_{a}^{\left(1\right)}+T_{a}^{\left(2\right)}=\frac{C_{a}p}{2}\pm \frac{C_{a}p}{2}\sqrt{1+\frac{4}{\tau_{a}^{2}}\frac{1}{p^{2}}}.$$

The first part is the basic differential operator, which is the common differential term in the response control equation. The second part is the multiplication of the ordinary differential operator *p* and the integral operator $\sqrt{1+\frac{4}{\tau_{a}^{2}}\frac{1}{p^{2}}}$, which is the modulation term in the control equation and called the modulating integral operator.

After binomial expansion and substitution of the convolution kernel function form of the integral operator according to the operator algebra operation rules [42], a special function is used to express the series formula. Finally, the modulating convolution kernel function form of the aortic FAO can be expressed as follows:

(27) $${\hat{T}}_{a}=\frac{C_{a}p}{2}\pm \langle \frac{C_{a}}{2t^{2}}\left(1-e^{-2t^{2}/\tau_{a}^{2}}\right)\rangle .$$

See Appendix A for the specific derivation process.

It is worth noting that, in operator algebra, the product symbol $\langle f(t)\rangle \cdot \langle g(t)\rangle$ of two functions is defined as the convolution of two functions, $\langle f(t)\rangle$ and $\langle g(t)\rangle$. They satisfy the following relation:

(28) $$\langle f(t)\rangle \cdot \langle g(t)\rangle ≜\int_{0}^{t}f\left(t-\tau \right)g\left(\tau \right)d\tau .$$

Based on general control Equation (15), kernel function form of FAO (27), and Equation (28), the convolution control equation of aortic FFC in the ISFCM can be uniformly expressed as:

(29) $$\langle i_{a}\left(t\right)\rangle ={\hat{T}}_{a}\langle u_{a}\left(t\right)\rangle =\langle \frac{C_{a}}{2}\left[\frac{du_{a}\left(t\right)}{dt}\pm \int_{0}^{t}\frac{1-e^{-2t^{2}/\tau_{a}^{2}}}{{\left(t-s\right)}^{2}}u_{a}\left(s\right)ds\right]\rangle ,$$

where the convolution kernel function of the modulation term of the aortic blood flow is:

(30) $$f_{a}=\frac{C_{a}\left(1-e^{-2t^{2}/\tau_{a}^{2}}\right)}{2t^{2}}.$$

It is worth noting that $f_{a}$ is a special Gaussian-type kernel function (GKF), which plays a central modulation role in aortic hemodynamics.

There are many types of GKFs. The basic form is $e^{-t^{2}}$, the common form is $1-e^{-t^{2}}$, $e^{-t^{2}}/t^{2}$, etc. Here, the kernel function in the aortic FAO also belongs to a kind of GKF called "Gaussian-type Kernel Function with Quadratic Quotient" (GKF-QQ). In order to facilitate the analysis of the intrinsic properties of the aortic kernel function, we simplify the physical parameters of the arterial vessels, taking $C_{a}=2cm\;\cdot mmHg^{-1}$ and $\tau_{a}=\sqrt{2}s$, then the GKF-QQ can be abbreviated as:

(31) $$f_{a}=\frac{1-e^{-t^{2}}}{t^{2}}.$$

**Figure 6:**
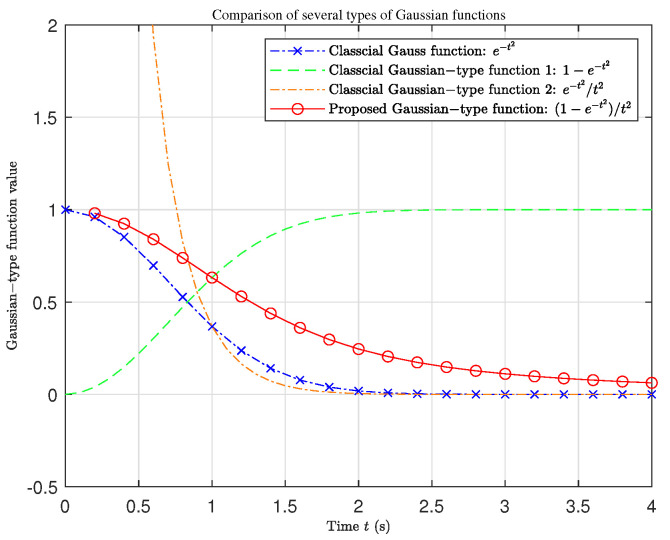
Comparison of four classes of Gaussian-type kernel functions.

The time-history behavior curves of four typical GKFs are compared, as shown in Figure 6.

It can be seen that GKF-QQ has a very high similarity with basic Gaussian function. They both approach 1 near 0, $\lim_{t\to 0}e^{-t^{2}}=1$ and $\lim_{t\to 0}\left(1-e^{-t^{2}}\right)/t^{2}=1$; As time approaches infinity, they both approach 0, $\lim_{t\to \infty }e^{-t^{2}}=0$ and $\lim_{t\to \infty }\left(1-e^{-t^{2}}\right)/t^{2}=0$. The inflection point of the basic Gaussian function is at t≈0.7 s, while the inflection point of the GKF-QQ is at t≈0.9 s, which is slightly larger than that of the basic Gaussian function. Moreover, compared with the basic Gaussian function, the GKF-QQ has a more gentle decreasing range and tends to zero longer. This means that the effective control time for blood flow of the artery is longer and the hysteresis effect is more clear. Such gentle decline and long hysteresis effect are the basic characteristics of general physiological blood flow. Therefore, GKF-QQ is a suitable theoretical tool to describe aortic hemodynamics.

### 3.4. Bessel-Type Convolution Modulation Kernel Function in Arteriolar FAO of ISFCM

Similar to the derivation of the aorta, the kernel function of arteriolar FAO can be obtained. The arteriolar FAO (Equation (17)) is decomposed into the sum of the basic term and the modulation term:

(32) $${\hat{T}}_{ar}=T_{ar}^{\left(1\right)}+T_{ar}^{\left(2\right)}=\frac{C_{ar}p}{2}\pm \frac{C_{ar}p}{2}\sqrt{1+\frac{4}{\tau_{ar}}\frac{1}{p}}.$$

After binomial expansion and substitution of the convolution kernel function form of integral operator [42], the other special function is used to express the series formula. Finally, the modulating convolution kernel function of arteriolar FAO is expressed as follows:

(33) $${\hat{T}}_{ar}=\frac{C_{ar}p}{2}\mp \langle \frac{C_{ar}e^{-\frac{2t}{\tau_{ar}}}}{\tau_{ar}^{2}}\left[{\bar{J}}_{0}\left(\frac{2t}{\tau_{ar}}\right)-{\bar{J}}_{2}\left(\frac{2t}{\tau_{ar}}\right)\right]\rangle .$$

Moreover, by combining Equations (15) and (33), the convolutional hemodynamic response control equation of arteriolar FFC in the ISFCM can be uniformly expressed as:

(34) $$\begin{array}{cc} \langle i_{ar}\left(t\right)\rangle & ={\hat{T}}_{ar}\langle u_{ar}\left(t\right)\rangle \\ \; & =\langle \frac{C_{ar}du_{ar}\left(t\right)}{2dt}\mp \int_{0}^{t}\left\{\frac{C_{ar}e^{\frac{-2(t-s)}{\tau_{ar}}}}{\tau_{ar}^{2}}\left[{\bar{J}}_{0}\left(\frac{2(t-s)}{\tau_{ar}}\right)-{\bar{J}}_{2}\left(\frac{2(t-s)}{\tau_{ar}}\right)\right]u_{ar}\left(s\right)\right\}ds\rangle . \end{array}$$

where the integral kernel function modulating flow behavior of the arteriole is:

(35) $$f_{ar}=\frac{C_{ar}e^{-2t/\tau_{ar}}}{\tau_{ar}^{2}}\left[{\bar{J}}_{0}\left(2t/\tau_{ar}\right)-{\bar{J}}_{2}\left(2t/\tau_{ar}\right)\right].$$

It can be seen that the zero-order and the second-order Modified Bessel Function of the First Type play the core modulation role in the response of the arteriolar FFC. Notice that this equation provides a quantitative dynamic mechanism for the study of blood flow behavior.

It is worth noting that the Bessel function is also a special kind of function, which has been widely used in many disciplines, such as the solution of Laplace equation in cylindrical coordinates, electromagnetic wave propagation in the cylindrical waveguide of wave mechanics, heat conduction law in cylindrical body, etc. Here, Bessel function also plays an important role in arteriolar hemodynamics. However, arteriolar Bessel kernel function has its own characteristics. It contains two parts. One is negative exponential function $C_{ar}e^{-2t/\tau_{ar}}/\tau_{ar}^{2}$. The other is Modified Bessel function $\left[{\bar{J}}_{0}\left(2t/\tau_{ar}\right)-{\bar{J}}_{2}\left(2t/\tau_{ar}\right)\right]$. Note that the Modified Bessel function is the “core”, and the exponential function is the weight factor. Therefore, we term the kernel function on arteriole as “Bessel-type Kernel Function weighted by Negative Exponential Function” (BKF-NEF). In particular, there are limitation features of the BKF-NEF: $\lim_{t\to 0}f_{ar}=C_{ar}/\tau_{ar}^{2}$, $\lim_{t\to \infty }f_{ar}=0$. It is similar to the classical exponential function $e^{-t}$, but the modulation amplitude is smaller and slower. Furthermore, it has similar behavior to the GKF of the aorta. We will discuss the similar and different properties of the two types of kernel functions in detail in the following Section 3.5.

### 3.5. The Differences and Similarities between GKF-QQ and BKF-NEF with Different Physical Parameters in Regulating Arterial Blood Flow

It is known that the physical characteristics of the vessels are variational. Differences exist between pathological and normal physiological states, between different organisms, and even between different segments of blood vessels within organisms. Therefore, it is necessary to discuss the regulation effect of ISFCM on blood flow under different physical parameters (characteristic time, inertia, viscosity, or compliance) in different physiological states.

**Figure 7:**
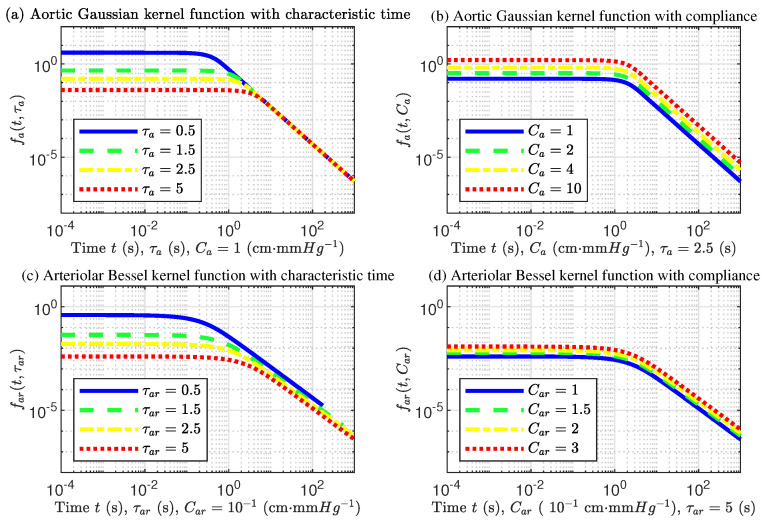
Aortic Gaussian-type kernel function with quadratic quotient (GKF-QQ) and arteriolar Bessel-type kernel function weighted by negative exponential function (BKF-NEF) modulated by different characteristic time and vascular compliance, respectively: (**a**) Aortic GKF-QQ changes with characteristic time; (**b**) Aortic GKF-QQ changes with vascular compliance; (**c**) Arteriolar BKF-NEF changes with characteristic time; (**d**) Arteriolar BKF-NEF changes with vascular compliance.

If the compliance is taken as a constant, $C_{a}=1$ cm · mm$Hg^{-1}$, in the aortic modulation kernel Function (30), the aortic modulation kernel function will change with the characteristic time, as shown in Figure 7a where only the aortic flow inertia needs to be changed. On the other hand, if the characteristic time of the aorta is kept constant (for example, selecting the value at the ascending aorta, $\tau_{a}=2.5$ s), the GKF-QQ changes with the vascular wall compliance, and its curve is shown in Figure 7b.

Similarly, in the arteriolar kernel function Equation (35), if the compliance is taken as a constant (for example, at arm arteriole, $C_{ar}=0.1$ cm · mm$Hg^{-1}$), the image of the change of the kernel function with the characteristic time is shown in Figure 7c where only the blood viscosity of the arteriole can be changed. If the characteristic time of the arteriole remains unchanged (for example, in the arteriole of the arm, $\tau_{ar}=5$ s), the curve of BKF-NEF in arteriole changes with respect to vessel wall compliance, as shown in Figure 7d.

It can be found that although the modulated integral kernel functions of the aorta and arteriole are different in form, they have similar modulation trends, which can be divided into two main stages: stability and power exponential attenuation regulation. The trend changed with the characteristic time, and wall compliance being the same in the two kernel functions. The characteristic time can change the critical moment at the inflection point of the blood-flow function, but the compliance only affects the blood-flow amplitude.

As shown in Figure 7a,c, if the characteristic time (blood inertia or viscosity) is increased, the short-term regulatory effect of the artery is increased, but the amplitude is decreased, while the long-term decaying exponent will be kept constant. This means that when it is necessary to reduce blood flow, the value of blood inertia or blood viscosity parameter needs to be increased, while keeping the wall compliance unchanged. Moreover, to maintain a constant long-term attenuation index, constant vascular compliance is required.

On the other hand, as shown in Figure 7b,d, keeping the characteristic time of aorta and arteriole unchanged, GKF-QQ and BKF-NEF increased with the aortic and arteriolar wall compliance, respectively. But the critical inflection point is unchanged. This suggests that when regulating parameters in disease treatment, such as atherosclerosis and blood flow mimicry in vitro, we only need to change the wall compliance to adjust the amplitude of arterial blood flow.

However, there are also differences between the two functions. At the turning point of sharp decline, GKF-QQ of aorta changes more suddenly and violently than BKF-NEF of arteriole, while the change of arteriole is relatively gentle. It is caused by the viscous dissipation in arteriole.

The modulation kernel functions of the aorta and arteriole not only show the heterogeneity but also show the correlation of the biological arterial system. If we want to use in vitro mimic methods to control the physical characteristics of the artery and accurately change the arterial blood flow state, the ISFCM can intuitively achieve the desired results.

### 3.6. Blood Flow Response Modulated by GKF-QQ and BKF-NEF of Aortic and Arteriolar ISFCM Respectively

This section focuses on the response of arterial hemodynamics modulated by GKF-QQ and BKF-NEF, respectively, and also discusses the sensitivity of the ISFCM to parameters.

The input of the arterial blood-pressure pulsation selected is the simple harmonic pulse wave signal. It is helpful to discuss the effect between the blood-flow response signal and the blood-pressure pulsation input signal when modulated by the GKF-QQ and BKF-NEF with different physical parameters in the aorta and arteriole, respectively. In physiology, the propagation process of blood pressure pulsation is called pressure pulse wave. A complete pressure pulse wave can be regarded as the superposition of the primary pulse wave caused by systolic and diastolic pulsation and the repulse wave caused by wave reflection. Therefore, we assume that the complete pulse wave consists of the superposition of two sinusoidal harmonics and the period of the pulse wave is approximately equal to the cardiac period $t_{0}=0.8$ s,

(36) $$P\left(t\right)=P_{1}\left(t\right)+P_{2}\left(t\right)=\left\{\begin{array}{c} P_{0}+P_{0sin}\left[\sin(3\pi t/t_{0}-\pi /2)+1\right],0⩽t⩽t_{0}/3;\\ 2P_{0}+P_{0sin}[\sin\left(3\pi t/t_{0}-\pi /2\right);\\ +k\sin\left(3\pi t/t_{0}-3\pi /2\right)+k+1],t_{0}/3⩽t⩽2t_{0}/3;\\ P_{0}+kP_{0sin}\left[\sin(3\pi t/t_{0}-3\pi /2)+1\right],2t_{0}/3⩽t⩽t_{0}. \end{array}\right.$$

Here, in the aortic blood pressure pulsation wave $P_{a}\left(t\right)$, take $P_{0a}=22$ mmHg, $P_{0sina}=20$ mmHg, $k_{a}=0.7$. In the blood pressure pulsation wave $P_{ar}\left(t\right)$ of arteriole, $P_{0ar}=20$ mmHg, $P_{0sinar}=35$ mmHg, $k_{ar}=0.5$. They are generally representative parameters that approximate realistic blood flow based on the data presented by Olufsen [44] and O’Rourke [45], where Olufsen does not specify exactly how the data were measured and O’Rourke adopts non-invasive experimental measurement methods. Notice that these data can also be obtained by non-invasive experimental cases, X-ray [46,47], or ultrasound [48].

Since arterial compliance mainly affects the amplitude of blood flow, we temporarily ignore the changes in blood flow regulation caused by the compliance and assume that they are constant values, $C_{a}=1$ cm· mm$Hg^{-1}$ and $C_{ar}=0.1$ cm· mm$Hg^{-1}$, respectively. Here, we focus on the changes in the behavior of regulated blood flow of the aorta and arteriole, when the characteristic time $\tau_{a}=\sqrt{LC_{a}}$ and $\tau_{ar}=RC_{ar}$ become the adjustable parameters with the change of blood inertia and viscosity, respectively.

**Figure 8:**
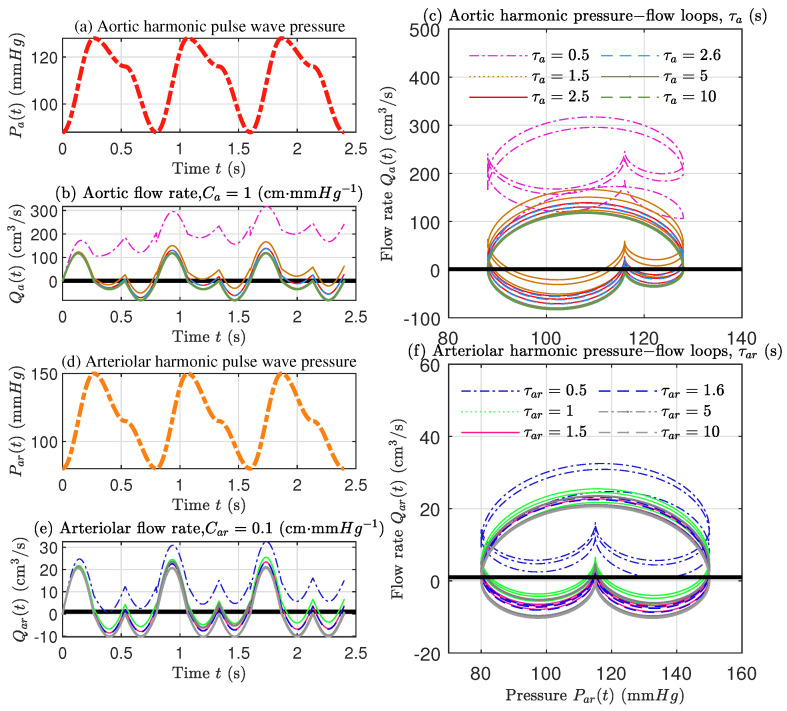
The responses of arterial blood flow modulated by GKF-QQ and BKF-NEF of ISFCM with harmonic blood-pressure pulsation input waves. (**a**) Aortic harmonic blood-pressure pulse wave input signal; (**b**) Blood-flow time-varying response of aorta modulated by GKF-QQ with different characteristic time $\tau_{a}$; (**c**) The associated hysteresis loop of aortic blood flow and blood pressure with different $\tau_{a}$; (**d**) Arteriolar harmonic blood-pressure pulse wave input signal; (**e**) Blood-flow time-varying response signal modulated by BKF-NEF of arteriole varying with different characteristic time $\tau_{ar}$; (**f**) Associated hysteresis loops of arteriolar blood flow and blood pressure with different $\tau_{ar}$.

After inputting the harmonic blood-pressure pulsation signal, the corresponding blood flow signals modulated by GKF-QQ and BKF-NEF will change with different characteristic times. The blood flow pulse pressure input, blood flow rate response, and corresponding flow–pressure hysteresis loop curves are all shown in Figure 8. Here, the modulation term of the response control equation is selected as the “positive effect”, that is, the ”+” modulation term is used in the expression of the aortic FAO. Whereas, the FAO of arteriole takes the modulation term as ”−”.

With the input of harmonic pulse pressure wave as shown in Figure 8a,d, aortic and arteriolar output waveforms both have negative blood flow, which shows the reflux phenomenon (see Figure 8b,e). It appears with the re-pulsation wave of blood pressure. The responses are consistent with the reverse flow signal measured by Marey’s early invasive method in 1881 using pitot tubes and needle flowmeters [49]. However, our simulated results are also consistent with the pressure wave reflection signals obtained by the noninvasive blood pressure measuring methods (by brachial artery cuff, electromagnetic technique, ultrasound (Doppler), cardiac magnetic resonance) summarized by O’Rourke et al. [38,45]. In addition, there is a phase difference of peak value between blood flow and blood pressure signal within a period, which means that the modulation of blood pressure and blood flow by arterial FAO has a certain time-delayed effect. As shown in Figure 8c,f, under the regulation of GKF-QQ and BKF-NEF, both aorta and arteriole show the hysteresis loop phenomenon, and the two curves have the similar shape and same time-delayed regulation effect. The loopback curve of ISFCM is matched with the structure tree model [44,50] and the measured loopback curve of the organism [43].

As we can see, both aortic and arteriolar models have stably regulated effective characteristic time range. For the aorta, with the increase of characteristic time, the flow response gradually tends to be stable, and its effective stable blood flow modulation interval is $\{\Omega_{a}:\tau_{a}\in [2.5$ s,+∞)}. For arteriole, it is $\{\Omega_{ar}:\tau_{ar}\in [1.5$ s,+∞)}. In comparison, the critical characteristic time of effective stable modulation interval of arteriole is smaller than that of the aorta. The critical characteristic time of arteriole is smaller, and the stable regulation range is wider. It is worth noting that for different input waveforms, the effective stable modulation interval of the ISFCM is not a fixed value. It will change with different input waveforms and related to the set percentage of critical flow difference, namely, the accuracy.

In addition, it can be found from Figure 8b,e that with the increase of blood characteristic time, the blood flow rate of both aorta and arteriole modulated by the ISFCM decreases. Here, vascular wall compliance is maintained constant, with characteristic time increase with blood inertia or viscosity. The features are consistent with the conclusion in Section 3.5. Therefore, in order to maintain fluent blood flow, it is necessary to keep low blood inertia and viscosity to maintain low blood characteristic time. This can be utilized to treat diseases such as blood obstruction when the vascular wall compliance is unchangeable and atherosclerosis has formed.

## 4. Discussions

### 4.1. Advantages of the Proposed ISFCM in Mimic Function and Theoretical Approach

The proposed model has the functional advantages of revealing the relationship between the self-similar structures and the blood flow phenomena of reflux, hysteresis loops. The traditional random stacked finite order hemodynamic circuit model is either too simple to describe the reflux and hysteresis loops behaviors [20] or too complex to ignore the influence of structural form on these phenomena [22]. Compared to these models, the proposed model clearly demonstrates the structural regulation of blood flow function due to its infinitely ordered self-similarity.

On the other hand, compared with the traditional continuum mechanics method, this paper adopts a simpler and more systematic mechanical-electrical analogy method to simulate the dynamic behavior of blood flow with a circuit model. However, the classical hemodynamic circuit model is only a finite-level model and cannot approach the realistic blood flow state. Therefore, an infinite-level circuit model is constructively proposed to simulate realistic blood flow theoretically. Although the infinite-level model appears to be more complicated, we use the fractal hypercell theory and the fractal admittance operator to simply and directly clarify the modulation mechanism of the model on blood flow. Further analysis of the fractal admittance operator shows that it has a convolution modulated form for blood flow and has Gaussian and Bessel-type kernel functions in aorta and arteriole segments, respectively. We found that by simply adjusting the parameters of fractal admittance operator and kernel function, such as characteristic time, wall compliance, blood inertia, and viscosity, the total flow output of vascular segment changes could be obtained directly. The simulations of kernel function and harmonic pulse signal show that low vascular wall compliance or high blood inertia and viscosity will lead to low blood-flow rate. Under the adjustment of the infinite model, we also reveal the realistic blood-flow phenomena of the arterial blood backflow and hysteresis loop. In a word, the ISFC model has the advantage of mimicking realistic blood flow in theory.

### 4.2. Origins of Structural Forms of the Convolutional Hemodynamic Modulation Mechanism

The inspiration stems from the research developed by Guo et al. [14,15] in tendons, ligaments, and nerve fibers. They [14] derived the governing equation of viscoelastic mechanical response of ligament fibers, based on the FHC element and operators with symmetry broken. They found that the equation contains the power-quotient exponential modulation kernel function. In addition, similar results can be obtained from the neural discharge of nerve fibers [15]. At the same time, it is proven that the power-quotient exponential kernel functions come from (a) the motion of fractal functional structure (or the motion on fractal structure) and (b) symmetry broken of fractal hypercell. It shows that there are exponential kernel functions on the symmetrically broken FHC.

It can be seen from the above analogy that the GKF-QQ (Equation (30)) and the BKF-NEF (Equation (35)), which are the convolutional kernel functions of arterial hemodynamic modulation mechanism, are generated under the conditions that: (a) the existence of fractal structure, i.e., infinite elastic cavity and its fractal circuit; (b) symmetry broken of fractal hypercell; (c) the basic physical elements of inertia, viscosity, and compliance present in the arteries. Among these three conditions, the fractal structure is the most basic. Therefore, the physical origins of GKF-QQ and BKF-NEF are fractal structures. In other words, the fractal structures cause the GKF-QQ and BKF-NEF of aorta and arteriole as modulated convolution core to effectively mimic the realistic blood flow, respectively.

### 4.3. Theoretical Revelation of the Proposed ISFCM for the Design of Bionic Materials with Self-Similar Structural Pattern

Traditional biomimetic materials for in vitro hemodynamics mimicry are based on the random stack of finite electrical components [22]. However, the self-similarity of the functional circuits which occurred naturally in arterial blood flow was ignored. The ISFC model provides a good chance to prepare biomimetic materials that can theoretically approximate realistic blood flow. According to the self-similar pattern of orderly assembly of electrical components, the bionic materials containing the ISFC can be designed and developed, which is known as self-similar biomimetic material (SBM). The SBM is a theoretical concept, and its possible utility is to mimic the physiological and pathological states of the vascular system in vitro, the same as ordinary hemodynamic simulation materials containing circuits [51]. Furthermore, the SBM will have important practical applications in the medical field, like that the theoretical model of cerebral arteriovenous malformation (AVM) hemodynamics will serve as a useful tool for theoretical investigations of AVM therapies and their hemodynamic sequelae [52]. With the SBM utilized to analyze the laws of arterial blood-flow dynamics in vitro, it is helpful to enhance the quantitative controllability, more easily and effectively explore the causes of diseases than that in vivo or the existing simulation materials in vitro, and provide structural pattern revelation for the development of new clinical medical schemes in the early prevention, diagnosis, and intervention treatment of diseases, such as atherosclerosis, as mentioned in Section 3 or shock, discussed in Section 4.4.

### 4.4. Feasibility of Shock Disease Study Based on ISFCM

In addition to atherosclerosis, shock (distributive shock, hypovolemic, etc.) is also an important cardiovascular disease. Shock is a comprehensive syndrome of systemic microcirculation dysfunction and serious impairment of vital organs, which is caused by the sharp decrease of effective circulating blood volume. As a result of the mechanism that the ISFC model can carry out convenient parameter regulation to simulate the pathological states such as the decrease of effective cardiac stroke volume of aorta and reduction of microcirculatory blood flow of arterioles, the ISFCM and its in vitro bionic materials are also effective in predicting blood-flow-reducing diseases such as shock. Specifically, when the pathogenic factors lead to a sharp decrease in arterial compliance or a rapid increase in blood inertia and viscosity, the fractal admittance operators given by the ISFC models can directly regulate and predict the sharply reduced blood-flow output at the aorta and arteriole segments, which ultimately leads to a sharp decrease in effective circulating blood volume and shock. Unfortunately, the model proposed in this paper does not involve the blood-flow regulation behavior of the venous segment, so it cannot give a theoretical prediction of the blood flow in the systemic blood circulation of the whole body.

### 4.5. Feasibility of Selecting Finite Components Retaining the Self-Similar Structure Form for the Actual Preparation of Self-Similar Biomimetic Hemodynamic Materials Due to the Inability of Infinite Components

According to the infinite-level SFCM, the FAO can determine the realistic blood-flow state theoretically. However, when fabricating circuit components to design the SBM, it is impractical to assemble infinite-level self-similar circuits. Fortunately, Guo et al. [14] explored the structural stiffness of self-similar ligaments and tendons. It shows that if the stiffness $T_{1}=T_{2}$, the smallest representative hypercell in the self-similar structure only needs to be repeated in the fourth order, and its stiffness is close to the infinite-level self-similar structure. There is only a relative error e=0.02% between them. Hence, we merely need to retain the self-similar structural patterns of the model. For the preparation of the SBM, we can make the finite-level self-similar functional circuit model (FSFCM) just with four duplication times of hypercell to achieve the results close to the infinite-level SFCM, which has theoretically equivalent realistic blood vessels.

### 4.6. Long-Term Goals

Since we have only carried out a theoretical analysis on the model, our long-term goal will be to prepare bionic materials with self-similar structure circuits through experiments, and explore the experimental blood-flow phenomenon simulated by such bionic materials. In addition, this paper only studies the aortic and arteriolar simple models containing two types of electrical components. As blood-flow behavior is often the result of joint action by a variety of electronic components, we will increase the types of electrical components in the circuit model and establish a more detailed blood-flow simulation modeling based on the self-similar structural mode in the follow-up study. Since only general blood-flow parameters are selected for analysis in this paper, the comparison of the difference between normal physiological state and pathological state of blood flow will also be the subject of detailed discussion in subsequent studies.

## 5. Conclusions

Based on the mechanical-electrical analogy method, we propose the infinite-level self-similar functional circuit models (ISFCM) to theoretically mimic the aortic and arteriolar realistic blood flow. It is a self-similar functional circuit, which can be utilized in biomimetic materials for medical detection or cure, especially atherosclerosis and shock. We put forward the theoretical analysis of the blood-flow regulation mechanism of the ISFCM. We found that the blood-flow response of ISFCM is determined by the simple fractal admittance operator (FAO), which is derived from the minimum representative unit, termed fractal hypercell (FHC). Further analysis shows that FAOs have apparent fractional-order differential features. The aortic and arteriolar blood flow under the control of FAOs have convolution modulation forms with GKF-QQ and BKF-NEF, respectively. They are two different kinds of kernel functions, but the blood flow modulated by them both show the phenomenon of reflux and hysteresis loop, which are consistent with realistic physiological states. Studies indicate that the ISFCM with FAO can quantitatively regulate and effectively depict the response of blood-flow signal, which provides an available and convenient structural theoretical guidance tool for fabrication of in vitro biomimetic materials applied to medical detection or treatment.

## Figures and Tables

**Table 1:** Correspondence table of the analogy between electric and hemodynamic networks.

Hemodynamic	Electric	Quantitative Correlation
Pressure *P*	Voltage *U*	P=U
Flow Rate *Q*	Current *I*	Q=I
Blood Inertia ρ	Inductance *L*	$L=c_{L}\rho /\left(\pi r^{2}\right)$ [11,38,39,40]
Wall Compliance *E*	Capacitance *C*	$C=3\pi r^{2}/\left(2Eh\right)$ [11,40,41]
Blood Viscosity μ	Resistance *R*	$R=8c_{R}\mu /\left(\pi r^{4}\right)$ [39,40]

## Data Availability

The data presented in this study are available on request from the corresponding author.

## Abbreviations

The following abbreviations are used in this article:

FCM	Functional Circuit Model
SFC	Self-similar Functional Circuit
SFCM	Self-similar Functional Circuit Model
ISFC	Infinite-level Self-similar Functional Circuit
ISFCM	Infinite-level Self-similar Functional Circuit Model
FFC	Fractal Functional Circuit
SBM	Self-similar Biomimetic material
FSFCM	Finite-level Self-similar Functional Circuit Model
FHC	Fractal Hypercell
FAO	Fractal Admittance Operator
GKF	Gaussian-type Kernel Function
GKF-QQ	Gaussian-type Kernel Function with Quadratic Quotient
BKF-NEF	Bessel-type Kernel Function weighted by Negative Exponential Function

## Appendix A. Derivation of Modulating Integral Kernel Function Expression of Aortic FAO

In order to clarify the kernel function form of the modulating integral operator $T_{a}\left(2\right)$, we separate the core part $T_{a-int}^{2}$:

(A1) $$T_{a}^{\left(2\right)}=\frac{C_{a}p}{2}T_{a-int}^{2}=\frac{C_{a}p}{2}\sqrt{1+\frac{4}{C_{a}L}\frac{1}{p^{2}}}.$$

Using binomial series expansion [42], the operator $T_{a-int}^{\left(2\right)}$ can be expanded as:

(A2) Ta−int(2)=∑n=0∞1/2n4CaLnl2n.

According to the operator algebra operation rules, the integral operator $l^{n}$ can also be written as the form of convolution kernel function [42]:

(A3) $$l^{n}=\langle \frac{t^{n-1}}{(n-1)!}\rangle ,n⩾1.$$

The symbol 〈·〉 represents the symbolic representation of a continuous function on an interval (0,∞), that is, 〈f(t)〉 is a continuous function, and the product of two functions is the convolution.

Therefore, the convolution kernel of $T_{a-int}\left(2\right)$ can be obtained by the kernel form of the integral operator as follows:

(A4) Ta−int(2)=∑n=0∞1/2n4CaLnt2n−1(2n−1)!.

Through deduction, the series in Equation (A4) can be expressed as a function, and the final expression of the convolution kernel function of the modulating integral operator is obtained:

(A5) $$T_{a-int}^{\left(2\right)}=\langle \frac{e^{-2t^{2}/\tau_{a}^{2}}-1}{t}+\frac{2\sqrt{2}}{\tau_{a}}\int_{0}^{\sqrt{2}t/\tau_{a}}e^{-s^{2}}ds\rangle ,$$

where, $\tau_{a}=\sqrt{C_{a}L}$.

Substituting Equation (A5) into Equation (A1), the convolution kernel function of the modulating operator of the aortic response control equation is in the form of:

(A6) $$T_{a}^{\left(2\right)}=C_{a}pT_{a-int}^{\left(2\right)}/2=\langle \frac{C_{a}\left(1-e^{-2t^{2}/\tau_{a}^{2}}\right)}{2t^{2}}\rangle .$$
